# Therapeutic effect of umbilical cord blood cells on spinal cord injury

**DOI:** 10.1002/ibra.12101

**Published:** 2023-05-01

**Authors:** Jun‐Yan Zhang, Z. Du Steven, Ke‐Hua Liao

**Affiliations:** ^1^ Department of Anesthesiology Southwest Medical University Luzhou China; ^2^ Department of Integrative Biology University of Wisconsin‐Madison Madison Wisconsin USA; ^3^ The Sixth People's Hospital of Chengdu Chengdu China

**Keywords:** cell therapy, cord blood cells, spinal cord injury, therapeutic effect

## Abstract

Spinal cord injury (SCI) is a nervous system disease characterized by sensory and motor dysfunction, axonal apoptosis, decreased vascular density, and inflammation. At present, surgical treatment, drug treatment, and cell therapy can be used. Surgical treatment can improve motor and independent function scores, and drug treatment can promote the recovery of neurons in the spinal cord, but only improve symptoms. Complete recovery of SCI has not yet been achieved. However, the differentiation of stem cells brings hope for the treatment of SCI. Umbilical cord blood cells (UCBs) are ethically readily available and can repair neuronal damage. However, it is still unclear how they can improve symptoms and repair nerve severity. In this paper, the role of UCBs in the treatment of SCI is described in detail from different aspects such as behavior, morphology, and molecular expression changes, so as to provide new ideas and theoretical directions for future research.

## Graphical abstract

The treatment of cord blood cells for spinal cord injury can improve sensory and motor functions, which result in healing of the injured area and expansion of the hindlimb volume. Axonal regeneration, increase in glial cells, and release of trophic factors; angiogenesis and reduction of rupture; and decreased inflammation and changes in immune function can also take place. Neuronal regeneration leads to increased expression of the NeuN+ (neuron nuclear antigen‐positive), nerve growth‐promoting factor, the glial cell line‐derived neurotrophic factor, and so on, and apoptosis leads to the decreased expression of caspase‐3 and other molecules. Angiogenesis leads to the increased expression of the vascular endothelial growth factor, glial fibrillary acidic protein, and the von Willebrand factor. Inflammation is inhibited by increased interleukin‐10 and decreased tumor necrosis factor‐α.

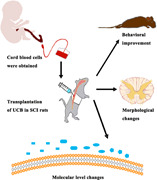

## 1 INTRODUCTION OF SPINAL CORD INJURY

### 1.1 Concept of spinal cord injury

Spinal cord injury (SCI) is a serious life‐threatening disease, which is characterized by complete or incomplete paraplegia/quadriplegia due to the nonregeneration of the spinal cord, resulting in different degrees of neurological dysfunction. SCI can be life‐threatening due to recurring complications such as pneumonia, complete bed rest, and urinary tract infections. Usually, people older than 70 years of age are the most susceptible to SCI.^1^, ^2^ The incidence of SCI is gradually increasing, and the severe exercise restriction and repeated complications make the situation worse. In addition, it also adversely affects patients physically and psychologically and poses a heavy burden on families and the labor market. It also seriously affects the normal day‐to‐day life of patients and their families, and places a huge burden on the whole society.

### 1.2 Treatment of SCI

At present, drug therapy, surgery, and cell transplantation strategies of neurotrophic factors (NTFs) are mainly used to treat SCI. For example, nerve growth‐promoting factor (NGF), brain‐derived neutral factor (BDNF), and neurotrophin‐3 can play a role in the axonal regeneration of different neuron groups in the spinal cord tract.^3^ Compared with nonsurgical treatment, surgery leads to greater improvement in the American Spinal Cord Injury Association motor score and the Functional Independence Measure Motor Score.^4^ In addition, there are some other methods to treat SCI. The wheeled treadmill test shows the recovery of motor function after SCI.^5^ Through three‐dimensional bioprinting, a series of spinal cord biometric scaffolds are being developed to improve motor function after SCI.^6^ However, none of the above treatments can be used to completely treat SCI, and can only alleviate symptoms and complications. Stem cells exist in many different forms, including embryonic stem cells, induced stem cells, hematopoietic stem cells, urine stem cells, bone marrow mesenchymal stem cells (MSCs), umbilical cord MSCs, umbilical cord blood cells (UCBs), adipose‐derived stem cells, neural stem cells, and neuroepithelial stem cells. UCBs have gradually become an increasingly important source of transplantation because they can be collected in a noninvasive manner and due to ethical requirements, and have entered the early clinical trial stage.^7^ Stem cells are also being used to determine which types are more effective against SCI, for example, both human adipose tissue and UCBs have similar contributions to motor and sensory recovery after SCI via anti‐inflammation and improved axonal growth, although they show slight differences in cytokine and gene expression.^8^ At present, stem cells do not survive after transplantation, and mainly play a role in neuron recovery through exosomes. This article mainly focuses on the survival of UCBs after SCI, and the role of UCBs in different aspects such as behavior, morphology, and molecular expression changes in nerve and blood vessel recovery.

## 2 LITERATURE REVIEW OF THE UCB TRANSPLANTATION STRATEGY IN SCI

### 2.1 Method

The literature on cord blood cells and related nervous system diseases over the past 5 and 10 years was searched on and downloaded from PubMed. The keyword for cord blood cells is “(umbilical cord blood cells)”. The keyword for SCI is “(spinal cord injury) OR (Spinal Cord Injuries) OR (“Spinal Cord Injuries”) OR (spinal injuries)”.

### 2.2 Results

A total of 38,428 articles about UCBs were retrieved, of which 5393 articles were those carried out in the past 5 years and 11,172 articles were those carried out in the past 10 years. A total of 171 articles on UCBs and SCI were retrieved, of which 50 were carried out in the past 5 years and 100 were carried out in the past 10 years (Table 1).

**Table 1:** Literature search.

Keyword(s)	Recent 5 years	Recent 10 years	IF > 10	6 < IF < 10	All results
(umbilical cord blood cells)	5393	11,172	3545	2731	38,428
((spinal cord injury) OR (Spinal Cord Injuries)) OR ((“Spinal Cord Injuries”) OR (spinal injuries)) AND (umbilical cord blood cells)	50	100	6	5	171

Abbreviation: IF, impact factor.

## 3 SURVIVAL TIME OF TRANSPLANTED UCBs IN SCI

Transplanted cells survive in animals for a short time, and it can be determined if the surviving cells affect the treatment or its derivatives. UCB‐derived pluripotent stem cells (MSCs) appeared in the injured spinal cord after 1 week, but no longer appeared after 6 weeks.^9^ UCB‐MSCs were observed in SCI 4 weeks after transplantation. The study has also shown that they can survive, but do not differentiate; for example, cells transplanted with UCB‐CD34+ survived at least 3 weeks at the injured site, but did not differentiate into nerve cells.^10^ On transplantation of UCB‐monocytes (UCB‐MCs), it was found that they survived at the injured site for 6 weeks, and there was no evidence of cell differentiation.^11^ However, other studies have shown that transplanted cells can survive and differentiate for a period of time, and transplanted UCBs can differentiate into various cells such as astrocytes and nerve cells. Labeled transplanted cells were observed in the white matter 21 days after transplantation of UCB‐MCs transfected with the pBud‐VEGF‐FGF2 plasmid.^12^ Moreover, after the transplantation of UCBs that were labeled with bromodeoxyuridine (BrdU) and nestin antibodies in SCI rats, cells with BrdU and nestin immunoreactivity were detected in the spinal cord, and expressed glial fibrillary acidic protein (GFAP), which is the marker of astrocytes, and MAP‐2, which is the marker of nerve cells.^13^, ^14^ Generally, the survival time of transplanted UCBs and their derivatives in rats is very short, usually no longer appearing at most 6 weeks, and they may not differentiate after survival. Therefore, the transplantation of UCBs may not only lead to cell proliferation but also secretion of some factors to improve SCI. NGF was secreted in vitro after addition of unrestricted somatic stem cells (USSCs) isolated from human cord blood in the injured spinal cord.^15^ The USSC‐secretory group contains proteins involved in many relevant neuroregeneration biological processes, with secreted protein, acidic, cysteine‐rich (SPARC) and pigment epithelium‐derived factor (PEDF) significantly involved in USSC‐mediated neurite growth.^16^ More information on the treatment effect of transplantation of UCBs and their derivatives in SCI will be described in detail below.

## 4 BEHAVIORAL IMPROVEMENT IN SCI PATIENTS AFTER TRANSPLANTATION

### 4.1 Behavioral improvement in a basic test

**Figure 1:**
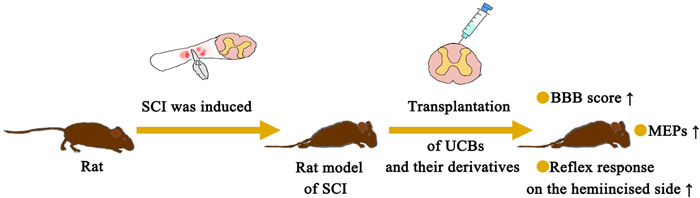
Behavioral changes after treatment of spinal cord injury (SCI). BBB, Basso, Beattie, and Bresnahan; MEPs, motor evoked potentials; UCBs, umbilical cord blood cells. [Color figure can be viewed at wileyonlinelibrary.com]

Transplantation of UCBs and their derivatives significantly improved motor function (Figure 1). It can be seen that the Basso, Beattie, and Bresnahan (BBB) scores and motor evoked potentials (MEPs) of SCI rats treated with UCBs improved, and the hindlimb dysfunction was obviously alleviated. This also confirmed the timeliness of using UCBs and their derivatives to treat SCI. First, on treatment with UCBs, an increase in the BBB score and recovery in hindlimb movement can be seen. More importantly, the proportion of rats with paralysis decreased from 70.8% to 20.8%, while the proportion of normal rats increased from 25% to 50%. The hemi‐incisal reflex response was improved after UCB transplantation.^17^, ^18^, ^19^ Then, UCB‐MSCs induced improvement at 2 weeks after transplantation. Hindlimb movement improved gradually at 3 weeks after transplantation, and BBB scores improved significantly at 6 weeks after transplantation.^9^, ^14^ Functional motor improvements of BBB, the Horizontal step walking test, and CatWalk gait analysis were observed after treatment with a well‐defined somatic stem cell from UCBs.^15^ Besides, the BBB size on injection of human UBC MCs was evaluated using the stress test and the open site test. The results showed that the motor recovery was good, and the motor function of the hind limbs improved obviously, which recovered from the two‐joint motor level to the three‐joint motor level.^11^, ^19^, ^20^ Moreover, transplantation of UCB‐CD34+ significantly improved BBB and Tarlov scores, promoted motor function recovery, and improved the survival rate.^10^ Platelet‐rich plasma from cord blood (UCB‐PRP), which includes cord blood cells and plasma, can also restore motor function when used alone as assessed from the BBB score.^21^ In addition, active motor recovery was targeted with a recombination gene. Motor function was improved on transplantation of UCB‐MCs containing the progenitor glial cell line‐derived neurotrophic factor (GDNF) mediated by the adenovirus (ADV) vector.^22^ There was limited improvement in knee function after transplantation of UCB‐MCs expressing recombinant genes VEGF165, GDNF, and neural cell adhesion molecule 1 (NCAM1).^23^ Adjunctive treatment with electrical stimulation also improved motor function in rats. The BBB score and MEPs increased after UCB‐MC transplantation combined with repetitive transcranial magnetic stimulation (rTMS).^24^ The motor function of SCI rats was improved by epidural stimulation combined with the inclusion of the vascular endothelial growth factor (VEGF) in UCB‐MCs.^25^ The motor function of SCI rats was improved on UCB‐MC transplantation combined with ultrashort wave therapy.^26^

### 4.2 Behavioral results of patients in clinical trials

Clinical trials showed no change in exercise, the SCI walking index (WISCI) and spinal cord‐independent measure (SCIM) scores in patients treated with UCB‐MCs for 12 months.^27^ However, studies have also shown that when patients are treated with UCB‐MCs for about 1 year (41–87 weeks), WISCI and SCIM scores increase, which can lead to improvements in walking, bladder and intestinal management, and recovery from complete sensory and motor loss to incomplete loss.^27^ Cord blood sample treatment applied to rhesus monkeys promoted obvious functional recovery of motor parts. However, a total of 419 adverse events were found, two of which may be related to cell therapy, but both were mild and clinically unimportant events.^28^

## 5 MORPHOLOGICAL CHANGES OF SCI AFTER TRANSPLANTATION

### 5.1 Macroscopic changes in morphology

A single intravenous injection of UCBs induced an increase in the volume of the hind limbs for 8 weeks.^17^ Damage decreased and tissue retention increased after treatment of a well‐defined somatic stem cell with UCBs.^15^ PRP injection of UCBs reduced the size of the lumen.^29^ Tissue was preserved on the 30th day after injury, and UCB‐MCs were transplanted to the lateral and ventral side of the umbilical cord 5 mm away from the center of the lesion.^30^ UCB‐MCs do not affect the expansion of lesions.^11^ On the 30th day after UCB‐MCs were transfected with VEGF and FGF2 genes, the total cross‐sectional area of the outer white matter cavity at 3 mm of the tail of the injury center decreased significantly.^31^ UCB‐MCs modified with VEGF and GNDF genes can induce tissue retention.^22^ UCB‐MCs, an ADV vector mediated by GDNF, were used to treat the preserved tissues in high areas.^32^ Epidural stimulation combined with VEGF in UCB‐MCs significantly reduced the morphological function of skeletal muscle of the hind limb of SCI.^25^ Transplantation of UCB‐CD34+ reduced the infarct size of the injured site.^10^

The tibialis anterior muscle was highly preserved and the H/M (monosynaptic reflex/motor response) ratio of gastrocnemius was increased, in addition to reduction of apoptosis and increased gray matter and white matter retention after the transplantation of UCB‐MCs expressing recombinant genes VEGF165, GDNF, and NCAM1.^23^ In addition, the lesion size of the UCB‐progenitor cell (PSC) treatment group is often smaller than that of the control group.^9^ Percutaneous transplantation of UCB‐PSCs reduced the size of cysts and lesions.^14^ In summary, with the transplantation of UCBs and their derivatives, the volume of injured sites such as spinal cord infarction and limb injury decreased. More importantly, we could observe an increase of myelinated fibers, growth of axons, regeneration of nerves, and a decrease in the number of fibroblasts.

### 5.2 Microscopic changes in morphology

Myelinated fibers were implanted 30 days after injury, and UCB‐MCs were implanted into the lateral and ventral cords 5 mm from the center of the lesion.^30^ Axon regeneration improved after treatment of a well‐defined somatic stem cell from UCBs.^15^ UCB‐CD34+ conditioned medium or 17β‐treated estradiol E2 and UCB‐CD34+‐conditioned medium (more prominently) significantly reduced the number of neuroapoptotic cells and astrocytes in the injured spinal cord.^33^ In the main corticospinal tract, UCB‐MCs transgenic with VEGF and GDNF significantly increased the number of spare myelinated fibers (22 times) compared with nontransgenic ones.^30^ Axonal regeneration of UCB‐MCs genetically modified with VEGF and GNDF genes was observed.^22^ ADV vector UCB‐MCs, mediated by GDNF, with a large number of standby myelinated fibers were used.^32^ UCB‐CD133+ resume axonal growth in coculture of inhibited cortical and spinal cord organs. UCB‐CD133+ can also reduce apoptosis.^34^ The number of myelinated fibers in UCBs transfected with a single PBUD‐VEGF‐FGF2 plasmid increased.^35^ On the 30th day after UCB‐MC transfection of VEGF and FGF2 genes, the number of myelinated nerve fibers increased in the white matter of the same region, at the same location of a cephalic and caudal distance from the source, and at 5 mm in the cephalic direction.^31^ SCI‐induced neuronal apoptosis was attenuated after UCB‐MC treatment combined with rTMS.^24^ There was a neuroprotective effect of UCB‐MC, derived nanovesicles, incorporated into macrophage plasma membranes by reduction of neuronal apoptosis.^36^

The improvement of the perineural environment is equally important for the repair of SCI. There were higher levels of synaptophysin, decreased numbers of astrocytes and microglia, and increased levels of Chrm1, as well as the muscarinic acetylcholine receptor M1, indicating higher levels of the excitatory neurotransmission system after epidural stimulation combined with VEGF in UCB‐MCs.^25^ Injection of PRP into UCB reduced the number of fibroblasts.^29^ HNA‐positive cells injected with UCB‐MC or UCB‐MC transgenic VEGF and GDNF have the morphology of phagocytes and microglia, and compact cell clusters or cell bridges are found in the wound cavity lined by astrocytes of the GFAP‐positive host.^30^ Transgenic UCB‐MC overexpressing NTFs increased the number of Schwann cells at the injured site.^37^ HUC‐MC transplantation combined with ultrashort wave therapy reduced the infiltration of CD3+ T cells and reduced the induction of microglia and A1 astrocytes.^26^

Myelinated fibers, axons, and nerves increased, while fibroblasts decreased, which increased blood vessel destruction. In addition, an increase in the number of perivascular cells expressing platelet‐derived growth factor β receptor (PDGFβR) could be seen. Transplantation of UCB‐CD34+ increases vessel density.^10^ UCB‐CD133+ cells also reduced the injury of cortical vessels.^34^ After 30 days of UCB‐MC transfection of recombinant VEGF and FGF2 genes, the number of perivascular cells expressing PDGFβR in the outer white matter increased by an average of 30%.^12^ Angiogenesis occurs with an increase in endothelial cell migration and tube formation after MC‐NV is incorporated into macrophage plasma membranes.^36^

### 5.3 Morphological changes in the clinical environment

The morphological changes in clinical experiments are closer to the real situation. Diffusion tensor magnetic resonance imaging showed the white matter space at the injured site before treatment in five patients.^27^ At the 12th month, fibrous bundle growth occurred in two patients, and the white matter space was smaller in other patients.^27^

Within 21 days after transplantation of UCB‐MCs transfected with recombinant vascular endothelial growth factor and growth factor 2 genes, epidermal growth factor protein (EGFP)‐labeled cells were traced in white matter at a location not less than 10 mm from the nearest rostral side and caudal side of injection. After 30 days of transfection of recombining VEGF and FGF2 genes with UCB‐MCs, the cross‐sectional area of gray matter retained at 3 mm/5 mm from the injury center increased by more than 60%.^12^

### 5.4 Summary of morphological changes

**Figure 2:**
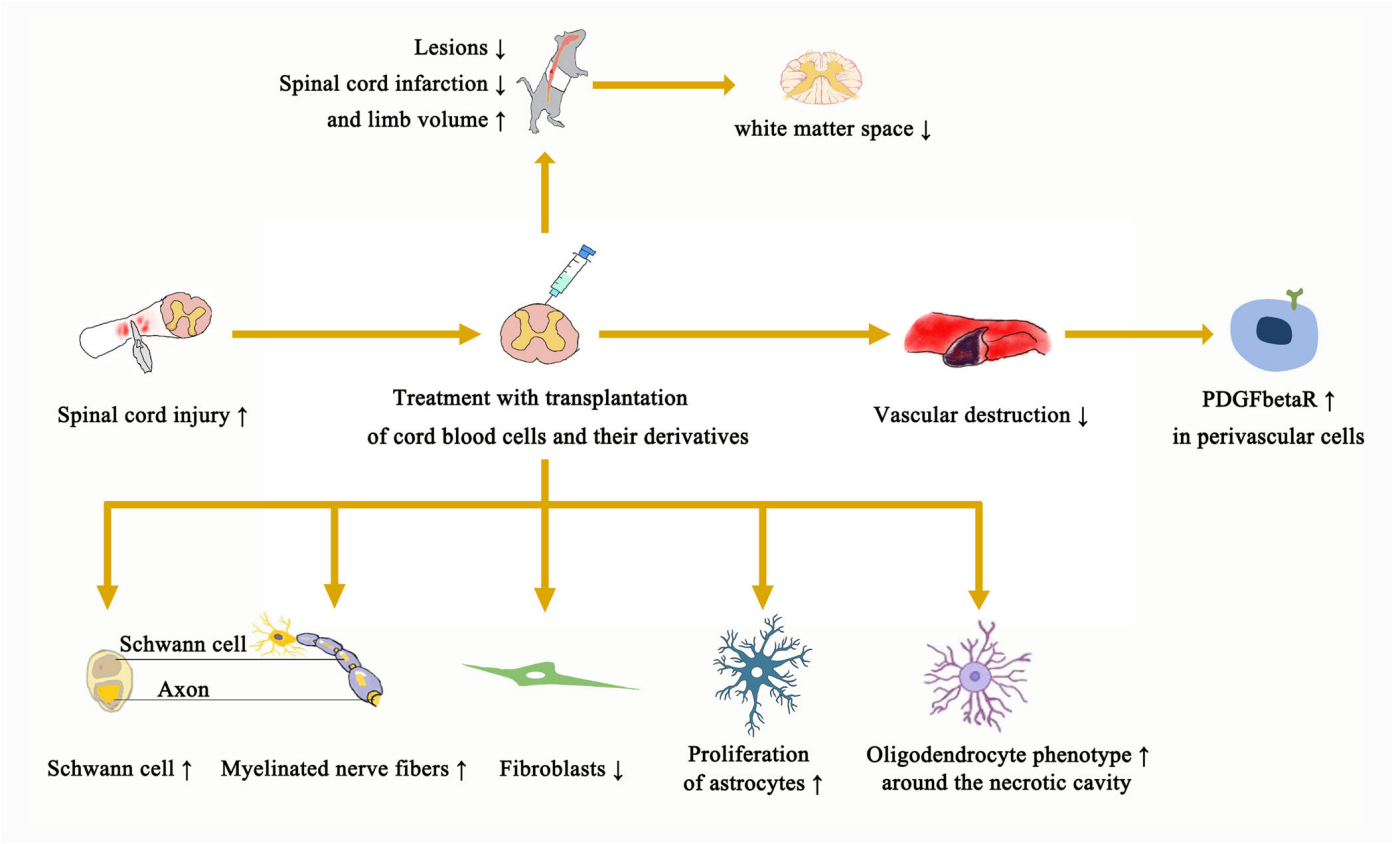
Morphological changes after treatment of spinal cord injury. PDGFβR, platelet‐derived growth factor β receptor. [Color figure can be viewed at wileyonlinelibrary.com]

Studies have shown that lesions and spinal cord infarction are reduced, but limb volume is increased. In addition, myelinated fibers, axons, and nerves increased, while the number of fibroblasts decreased. The number of apoptosis cells and astrocytes decreased, and the oligodendrocyte phenotype was observed around the necrotic cavity. It was found that the traumatic cavity with astrocytes was a compact mass or cell bridge. Vascular destruction decreased, and the number of perivascular cells expressing PDGFβR increased. The number of Schwann cells increased. Clinically, the white matter space narrows and the number of myelinated nerve fibers increases (Figure 2).

## 6 MOLECULAR‐LEVEL CHANGES DURING TREATMENT

### 6.1 Molecular nerve recovery

GDNF can be detected in the injured spinal cord after UCBC transplantation.^38^ NGF is also involved in this treatment. UCB‐PSCs were detected with an increased level of neuroprotection.^9^ UCB isolated from human umbilical cord blood has been found to have well‐known neuron growth‐promoting factors, such as neuron growth regulator 1, neuron‐derived neurotrophic factor, SPARC, and PEDF, which span the whole range of the USSC‐secreting group.^16^ Specific markers for UCB‐MCs with GDNF treatment are astrocytes of GFAP, serum 100β and aquaporin 4, oligodendrocytes of PDGFαR and Cx47, and Schwann cells of P0. The expression patterns of ADV‐GDNF and UCB‐MCs with ADV‐treated rats were different in different regions of the spinal cord.^32^ The microtubule system is a skeletal component of nerve cells, which can serve various cellular functions. Microtubules are composed of tubulin‐ and microtubule‐related proteins. Tau protein is the most abundant microtubule‐associated protein. After transplantation of PRP from human umbilical cord blood, the level of cerebrospinal fluid‐τ (CSF‐τ) decreased.^29^ After transplantation of platelet‐rich plasma from human umbilical cord blood, the level of myelin‐associated glycoprotein in the spinal cord increased.^21^

Neuron nuclear antigen (NeuN) is only expressed in mature neurons. Therefore, NeuN was also observed in UCB‐MSC transplanted cells.^14^ Combining retinoic acid and sonic hedgehog, the expression of motor neuron‐related markers increased after transplantation of motor neuron‐like cells derived from UCBs.^39^ GFAP is a marker of astrocyte activation. In addition, GFAP was positive in UCB‐MSC‐transplanted cells.^14^ The number of biotin‐glucanamide‐positive fibers and Brdu‐, nestin‐, and Tuj1‐positive cells in the corticospinal tract was significantly increased, while the number of Ng2‐ and GFAP‐positive cells was significantly decreased after UCB‐MC treatment combined with rTMS.^24^ Expression of miR‐9, miR‐let7b, miR‐137, and miR‐324‐5p, which are involved in neuronal differentiation and neuron/motoneuron maturation, was significantly increased after therapy with motor neuron‐like cells derived from UCBs. miR‐9‐5p and miR‐324‐5p, which are involved in neuronal cell and motoneuron differentiation, are upregulated in the early stages of differentiation. However, the expression of miR‐137 and miR‐let‐7b, considered to be regulators of cell proliferation, was downregulated at later stages of differentiation.^39^

### 6.2 Molecular growth and apoptosis

UCB‐PSCs were detected to have elevated levels of growth factors.^9^ The expression levels of basic fibroblast growth factor and EGF increased after UCB‐MC treatment combined with rTMS.^24^ Mammalian target of rapamycin (mTOR) is relatively conservative. It can integrate various extracellular signals, such as nutrition, energy, and growth factors, participate in biological processes such as gene transcription, protein translation, and ribosome synthesis, and play an extremely important role in cell growth, apoptosis, autophagy, and metabolism. The p‐mTOR/mTOR ratio was decreased by injecting PRP into cord blood.^29^ The level of caspase‐3 was decreased after transplanting PRP from human umbilical cord blood platelet‐rich plasma.^21^ In addition to regulating the activity of glycogen synthase kinase (GSK), GSK‐3β can also act on many signal proteins, structural proteins, and transcription factors to regulate cell differentiation, proliferation, survival, and apoptosis. After transplantation of PRP from human umbilical cord blood, the level of GSK‐3β decreased.^21^

### 6.3 Molecular changes in the surrounding environment of nerve cells

The expression of heat‐shock and synaptic proteins was increased after transplantation of UCB‐MCs expressing recombinant genes VEGF165, GDNF, and NCAM1.^23^ The anti‐inflammatory effect of polarized M1 to M2 macrophages was observed in vitro after adding UCB‐MCs incorporated into macrophage plasma membranes, which is closely related to the spinal cord repair mechanism.^36^ Elevated levels of cytokines with known anti‐inflammatory effects were detected in UCB‐PSCs.^9^ Interleukin‐10 (IL‐10), also known as IL‐10, is a cytokine involved in inflammation and immunosuppression. After UCB infusion, the level of IL‐10 in the serum increased.^38^ The production of proinflammatory cytokines IL‐1β and IL‐6 was reduced after UCB‐MC transplantation combined with USW.^26^ Tumor necrosis factor‐α (TNF‐α) is an adipokine produced by activated macrophages, which participates in the inflammation of the whole body and is also one of the cytokines that stimulate the acute‐phase response. In response to irritants (infectious sources or tissue damage), TNF‐α circulates throughout the body, changes the characteristics of vascular endothelial cells by activating neutrophils, regulates metabolic activities of other tissues, and has a destructive effect by inducing local coagulation. After UBC injection, the serum TNF‐α level decreased by 17. 5%.^38^

### 6.4 Molecules for improving endothelial cells

VEGF can be detected in the injured spinal cord after UBC transplantation.^38^ UCB‐PSCs have elevated levels of cytokines with angiogenic properties.^9^ Percutaneous transplanted UCB‐MSC cells were positive for the von Willebrand factor.^14^ CSF contains a class of cytokines that can selectively stimulate the proliferation and differentiation of hematopoietic stem cells and transform them into blood cells of a certain line. After transplantation of PRP from human umbilical cord blood, the expression level of CSF‐τ decreased.^21^

### 6.5 Others

A total of 1156 proteins of the USSC‐secreting group were isolated from human umbilical cord blood.^16^ PRP injection into UCB decreased the expression of P2X3R and increased the expression of P2Y4R.^29^ Novel microRNAs associated with cholinergic, hedgehog, mitogen‐activated protein kinase, and janus kinase–signal transducers and activators of transcription signaling pathways were detected after therapy with motor neuron‐like cells derived from UCBs.^39^

### 6.6 Summary of molecular changes

**Figure 3:**
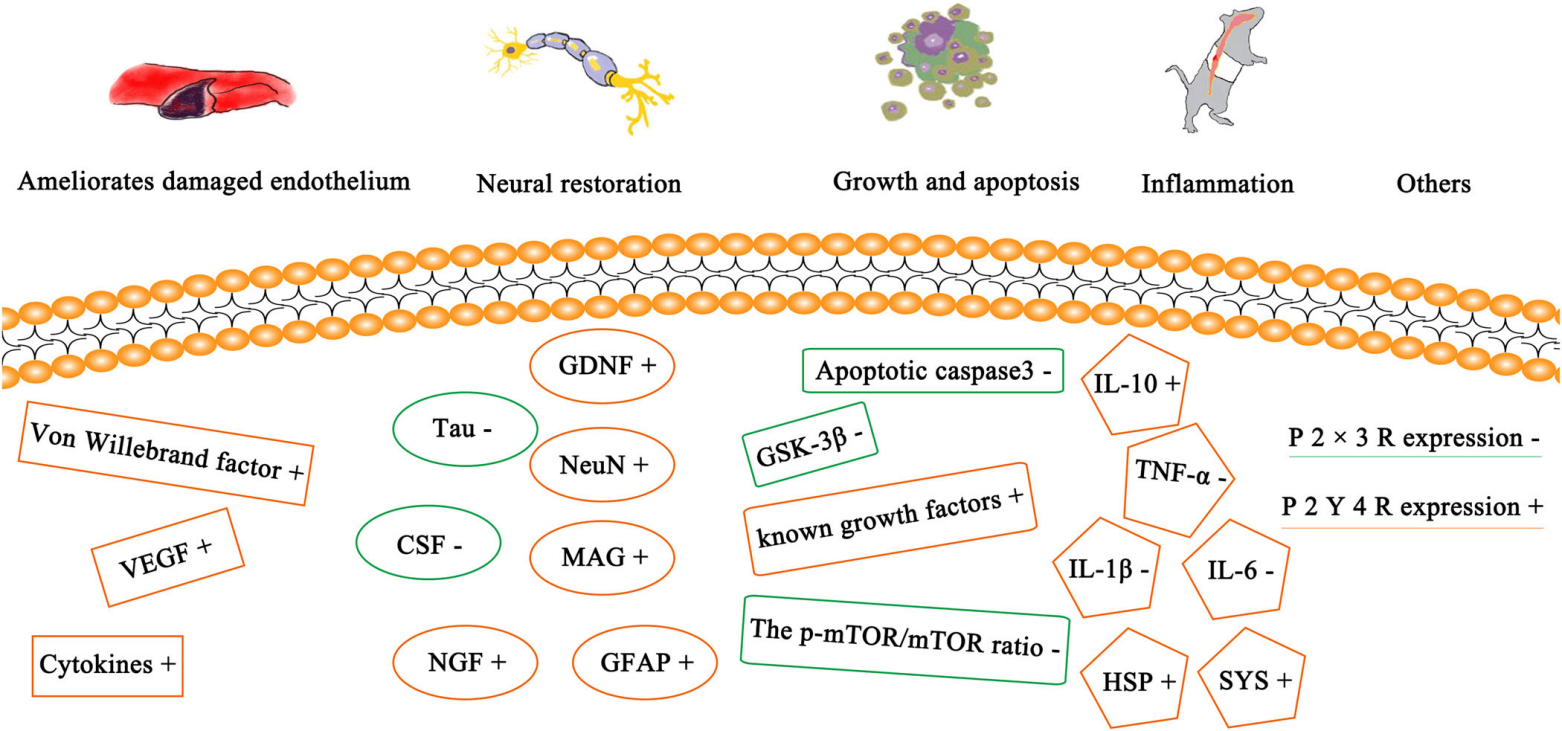
Molecular changes after treatment of spinal cord injury. CSF, cerebrospinal fluid; GDNF, glial cell line‐derived neurotrophic factor; GFAP, glial fibrillary acidic protein; GSK, glycogen synthase kinase; HSP, heat‐shock protein; IL, interleukin; MAG, myelin‐associated glycoprotein; mTOR, mammalian target of rapamycin; NeuN, neuronal nuclear antigen; NGF, nerve growth‐promoting factor; SYS, synapsin; TNF‐α, tumor necrosis factor‐α; VEGF, vascular endothelial growth factor. [Color figure can be viewed at wileyonlinelibrary.com]

Several studies have summarized different types of molecular changes after treatment with transplanted UCBs and their derivatives. These changes involve nerve recovery, endothelial improvement, growth and apoptosis, inflammation, and others revealing different expressions of the graft at the molecular level (Figure 3).

## 7 MECHANISM

SPARC is a glycoprotein that regulates the extracellular matrix and interacts with some matrix component growth factors and cytokines. SPARC participates in tissue remodeling, morphogenesis, cell migration and proliferation, and regulates physiological and pathological processes of the body. PEDF is a recently discovered factor that can effectively inhibit neovascularization. UCB is an unrestricted somatic cell isolated from human umbilical cord blood, and it has been confirmed that SPARC and PEDF are obviously involved in the growth of neuronal explants mediated by USSC.^16^

## 8 CONCLUSIONS AND PROSPECTS

UCBs can improve sensory and motor function in the treatment of SCI, which results in the reduction of the injured area and expansion of hindlimb volume, axon regeneration, improvement of glial cells and release of nutritional factors, reduction of angiogenesis and rupture, reduction of inflammation, and change of immune function. Neuron regeneration showed increased expression of NeuN+, NGF, and GDNF. In addition, apoptosis is characterized by decreased expression of caspase‐3 and other molecules. The expression of VEGF, GFAP, and Von Willebrand factor was increased in angiogenesis. Inflammation was inhibited by the increase of IL‐10 and the decrease of TNF‐α, IL‐1β, and IL‐6 (Table 2). However, the key molecular groups of UCB exosomes and their effects on nerve cell repair, apoptosis inhibition, and inflammation inhibition are still unclear. Future research should focus on finding key molecules to promote drug development and reduce host immune response after direct transplantation of UCBs, which will bring hope for the treatment of patients with SCI. Combined treatment is becoming increasingly more important and necessary, for example, UCBs combined with gene transfect and electric stimulation therapy for treatment of SCI.

**Table 2:** Therapeutic effect of UCB on SCI.

Site	Laboratory	Clinical
Survival time of UCBs	1–6 weeks^9^, ^10^, ^12^	
Behavioral improvement	Effective^9^, ^11^, ^14^, ^15^, ^17^, ^18^, ^19^, ^20^, ^21^, ^22^, ^23^, ^24^, ^25^, ^26^, ^38^	Effective^27^, ^28^
Morphological changes		
Spinal infarct	↓^9^, ^15^, ^17^, ^23^, ^25^, ^29^, ^30^	
Myelinated fibers	↑^15^, ^22^, ^29^, ^30^, ^34^, ^35^	↑^31^
Axons grow	↑^24^, ^25^, ^26^, ^33^, ^34^, ^36^	
Fibroblast numbers	↓^12^, ^34^, ^36^	
White matter space		↓^27^
Molecular‐level changes	p‐mTOR/mTOR−; P2X3R−; P2Y4R+^29^ GDNF+; VEGF+^38^ IL‐10+; TNF‐α−^38^ caspase3−; GSK3β−; CSF‐τ−; MAG+^21^ NeuN+^14^, ^39^ GFAP and vWF+^14^ NGF+^15^, ^16^, ^24^ IL‐1β−; IL‐6− ^26^ HSK and SYS+ ^23^	

Abbreviations: CSF, cerebrospinal fluid; GDNF, glial cell line‐derived neurotrophic factor; GFAP, glial fibrillary acidic protein; GSK, glycogen synthase kinase; HSP, heat‐shock protein; IL, interleukin; MAG, myelin‐associated glycoprotein; mTOR, mammalian target of rapamycin; NeuN, neuronal nuclear antigen; NGF, nerve growth‐promoting factor; PDGFβR, platelet‐derived growth factor β receptor; SYS, synapsin; TNF‐α, tumor necrosis factor‐α; UCB, umbilical cord blood cell; VEGF, vascular endothelial growth factor; vWF, Von Willebrand factor.

## AUTHOR CONTRIBUTIONS

Jun‐Yan Zhang organized, wrote, and revised this article. Z. Steven reviewed and collated the literature. Ke‐Hua Liao revised the whole article.

## CONFLICT OF INTEREST STATEMENT

The authors declare no conflict of interest.

## ETHICS STATEMENT

Not applicable.

## Data Availability

Data sharing is not applicable for this article because data sets have not been generated or analyzed in the current research.
